# Cochlear sparing in LINAC-based radiosurgery for vestibular schwannoma: a dosimetric comparison of dynamic conformal arc, IMRT and VMAT treatment plans

**DOI:** 10.1186/s13014-022-02188-y

**Published:** 2023-01-04

**Authors:** Jeremy Khong, Ramkumar Govindaraj, Daniel Ramm, Suzanne Edwards, Daniel Roos

**Affiliations:** 1 Department of Radiation Oncology, Royal Adelaide Hospital, Port Road, Adelaide, SA 5000 Australia; 2 School of Medicine, University of Adelaide, Adelaide, SA Australia; 3 Adelaide Health Technology Assessment (AHTA), School of Public Health, The University of Adelaide, Adelaide, SA Australia

**Keywords:** Cochlear sparing, Plan comparison, Stereotactic radiosurgery, Techniques, Vestibular schwannoma

## Abstract

**Background:**

Stereotactic radiosurgery (SRS) is the preferred treatment for vestibular schwannoma (VS) in patients with preserved hearing and tumour diameter < 3 cm. Emerging evidence suggests restricting cochlear dose could preserve hearing. This retrospective replanning study aims to compare dynamic conformal arc therapy (DCAT), intensity modulated radiation therapy (IMRT) and volumetric modulated arc therapy (VMAT) plans for superiority of cochlear dose sparing without compromising tumour coverage.

**Methods:**

Eligibility criteria included sporadic VS, serviceable hearing and availability of CT and MRI for planning. The original gross tumour volume and brainstem OAR volume were retained; the cochlea was newly contoured on the planning CT scan (bone window). Each case was replanned using the three above techniques, prescribing 12 Gy to the 80% isodose line. No dose constraint was applied to the cochlea.

**Results:**

Eighteen patients were replanned. Mean tumour volume was 2.25 cc. Tumour coverage and tumour mean dose (DCAT: 14.2, IMRT: 14.6, VMAT: 14.5 Gy) were comparable. Paddick and RTOG conformity indices were better for DCAT (0.66 and 1.6) and VMAT (0.69 and 1.5) compared to IMRT (0.56 and 1.9). DCAT had superior gradient index (3.0) compared to VMAT (3.4) and IMRT (3.4). VMAT delivered the lowest mean brainstem maximum dose (8.3 Gy) and decreased the mean cochlear dose (3.4 Gy) by 2.3 and 2.1 Gy, and the mean cochlear maximum dose (3.6 Gy) by 2.4 and 2.5 Gy relative to DCAT and IMRT, respectively.

**Conclusion:**

LINAC-based SRS treatment using VMAT can achieve better cochlear dose sparing than DCAT or IMRT while maintaining tumour coverage.

## Background

Vestibular schwannoma (VS) is a benign tumour arising from the Schwann cells of the vestibulocochlear nerve. It makes up 10% of all intracranial neoplasms and 80% of tumours at the cerebellopontine angle [1]. The early symptoms are hearing loss and tinnitus in the affected ear, but larger tumours may also be associated with gait imbalance. With improvements in screening for hearing loss and increased access to MRI, the incidence of VS is increasing [2] and these tumours are often detected while the patient still has useful hearing. Management options include observation, microsurgery or radiosurgery.

Stereotactic radiosurgery (SRS) has become the preferred treatment for VS in patients with preserved hearing and tumour diameter < 3 cm [3, 4]. Originally, SRS was performed using Gamma Knife (GK), but SRS can now be delivered using conventional LINAC or CyberKnife with parity in treatment outcomes [5, 6]. Moreover, LINAC-based SRS has grown technically more advanced with its ability to use intensity modulated radiation therapy (IMRT) and volumetric modulated arc therapy (VMAT) techniques, with the possibility of better sparing of organs-at-risk (OAR), especially the cochlea. As there is accumulating evidence supporting dose sparing of the cochlea to preserve hearing [7–11], choosing a LINAC-based treatment technique that is superior in reducing the cochlear dose is prudent. Moreover, dose sparing must be achieved without compromising tumour coverage, since limiting the dose to the cochlea at the expense of tumour coverage is currently not recommended [12, 13].

The purpose of this retrospective dosimetric study is to compare plans generated using dynamic conformal arc therapy (DCAT), IMRT and VMAT techniques for LINAC-based SRS of VS. The primary aim is to compare cochlear dose sparing achieved using these techniques without compromising tumour coverage. The secondary aim is to assess tumour-related factors that affect dose sparing of the cochlea.

## Methods

### Patient selection

Patients included in this treatment planning study were previously treated at the Royal Adelaide Hospital between 2000 and 2015. Selection criteria were unilateral VS, pre-treatment pure-tone average (PTA) less than 50 decibels (dB) and availability of CT and MRI for planning. Patients were excluded if they had bilateral VS (neurofibromatosis type 2), previous treatment with radiotherapy or surgery or received SRS for recurrent VS. Hospital Research Ethics Committee approval was obtained for this study.

### Planning methodology

**Fig. 1:**
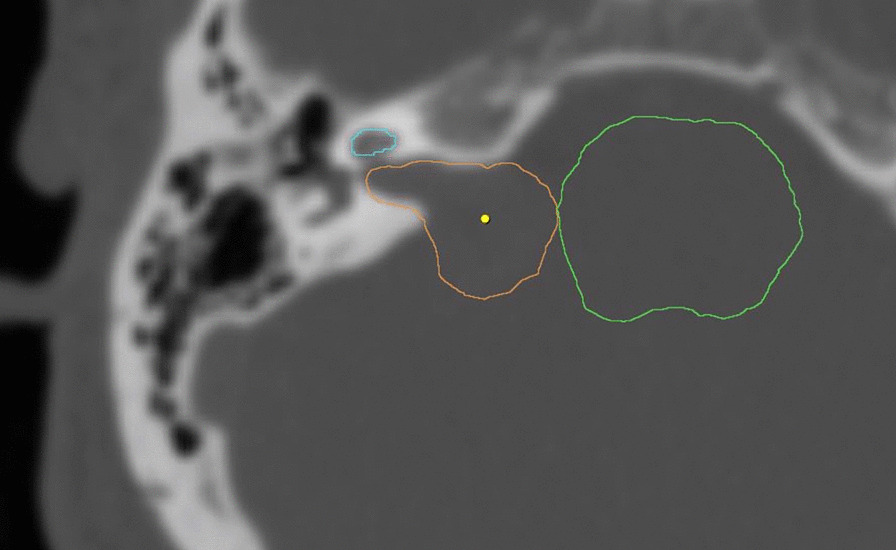
Example of contouring cochlea (light blue), GTV (orange), and brainstem (green)

**Fig. 2:**
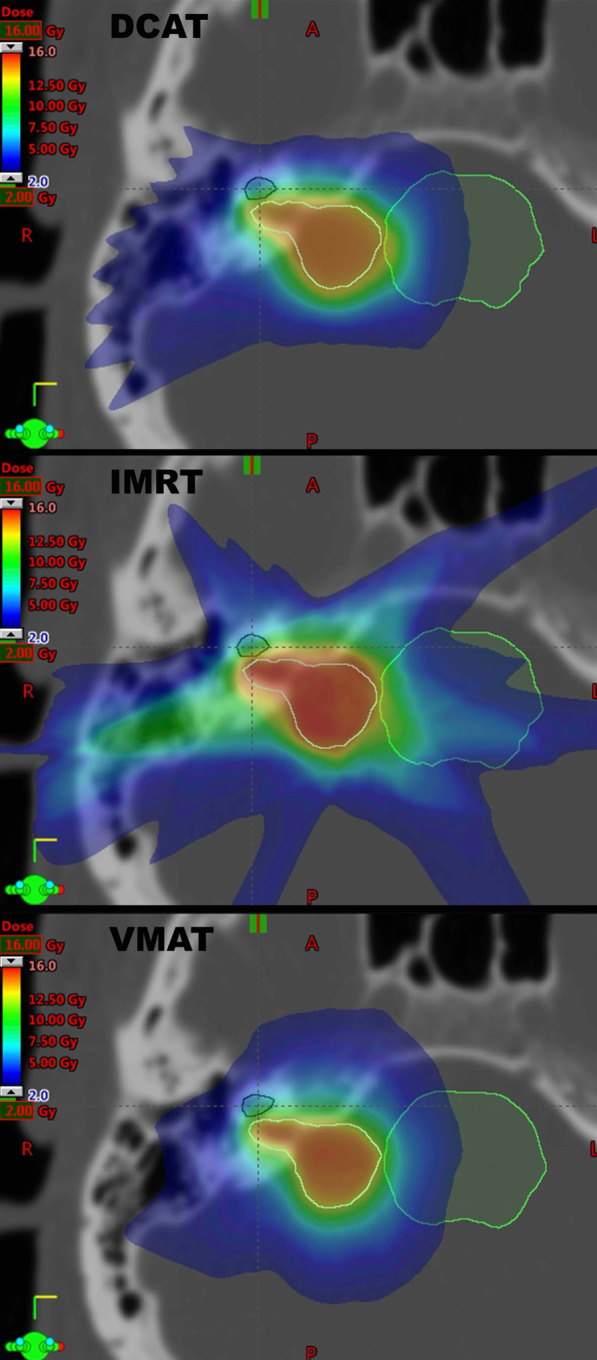
Isodose distribution for DCAT (top), IMRT (middle), VMAT (bottom) for a representative case. Yellow line = GTV; dark blue line = cochlea; green line = brainstem; PTV expansion = 0 mm*;* Colourwash threshold is set between 2 and 16 Gy

The original treatment plans of the selected patients were retrieved from the archive. The gross tumour volume (GTV) and brainstem OAR volumes were retained. As it was not a routine practice to contour cochlea in our institution, the cochlea was newly contoured using the bone window (3000–4500/600–800 HU) of the planning CT scan by two investigators independently [14] (Fig. 1). Any contour variation was assessed and resolved. No GTV to planning target volume (PTV) margin expansion was used. For each patient, the SRS was replanned using three techniques: DCAT (Brainlab iPlan RT 4.5), IMRT (Brainlab iPlan RT 4.5) and VMAT (Brainlab Elements RT Cranial SRS 1.5) (Fig. 2), prescribing 12 Gy to the 80% isodose line. The plans were accepted only if tumour coverage was > 99.9%. The IMRT and DCAT planning was performed by a senior radiation therapist and the VMAT by a senior radiation physicist who had no knowledge of the dosimetric outcomes of the IMRT and DCAT plans.

All treatment plans were generated using a Varian TrueBeam STx 6FFF beam model in the respective planning system. Both DCAT and VMAT plans were derived from standard non-coplanar 3 arc templates, with arc entry biased according to the laterality of the target. The Brainlab Elements VMAT planning system additionally provided an automatic arc angle optimisation driven by the locations of the PTV and OARs. The IMRT plans were generated using 9 beams, arranged according to target laterality. All plans were optimised for target coverage with sufficiently low priority on OARs to avoid compromising target coverage. Doses were computed using a 1 mm dose grid.

### Plan comparison

The plan quality was compared using the following dosimetric parameters: target coverage (D_98%_ and D_2%_), conformity index (CI) (RTOG, where lower is better, and Paddick, where higher is better), gradient index (GI, lower is better) and homogeneity index (HI) [15, 16]. OAR dose sparing achieved by each technique was compared using D_max_ (D_0.03cc_) and D_mean_ for the cochlea, and D_max_ (D_0.03cc_) and D_1cc_ for the brainstem. Tumour characteristics—volume, dimensions, distance from the fundus of the internal auditory canal and Ohata stage [17]—were also recorded to assess their association with cochlear dose.

### Statistical analysis

A linear mixed-effects model was employed to assess significant difference between the techniques for various dosimetric outcome parameters. A random effect was used to adjust for clustering on patient, as each patient underwent multiple techniques. Linear regression models were used to assess the association between various cochlear dosimetric parameters and tumour characteristics. The statistical software used was SAS 9.4 (SAS Institute Inc., Cary, NC, USA).

## Results

Eighteen eligible patients were identified. The mean tumour volume was 2.25 cc (SD 2.3), median 1.38 cc, range 0.34–7.7 cc. Other tumour characteristics are provided in Table 1, and descriptive statistics (plan quality parameters and OAR dose) in Table 2.

**Table 1:** Tumour characteristics

Characteristic	Mean (SD)
Volume (cc)	2.25 (2.3)
Diameter (mm)	
y	19.4 (4.4)
x	15.4 (6.7)
Distance from fundus (mm)	1.7 (1.9)

Ohata classification	Number of patients (%)
A	3 (16.7)
B	4 (22.2)
C	9 (50.0)
D	2 (11.1)

x = parallel to the axis of petrous bone in the axial plane; y = perpendicular to x

**Table 2:** Descriptive statistics of dosimetric parameters

Parameter (mean [SD])	DCAT	IMRT	VMAT
Conformity index			
RTOG	1.6 (0.2)	1.9 (0.4)	1.5 (0.2)
Paddick	0.66 (0.089)	0.56 (0.079)	0.69 (0.079)
Gradient index	3.0 (0.5)	3.4 (0.5)	3.4 (0.4)
Homogeneity index	1.3 (0.01)	1.3 (0.1)	1.3 (0.03)
Tumour			
D_mean_ (Gy)	14.2 (0.1)	14.6 (0.6)	14.5 (0.4)
D_98%_ (Gy)	13.0 (0.4)	13.4 (0.4)	13.2 (0.7)
D_2%_ (Gy)	15.1 (0.1)	15.3 (0.7)	15.5 (0.4)
Brainstem			
D_0.03cc_ (Gy)	10.3 (3.7)	9.0 (4.5)	8.3 (4.2)
D_1cc_ (Gy)	4.6 (2.5)	4.1 (2.6)	3.5 (2.2)
Cochlea			
D_0.03cc_ (Gy)	6.0 (1.8)	6.1 (2.5)	3.6 (1.8)
D_mean_ (Gy)	5.7 (1.6)	5.5 (2.3)	3.4 (1.7)

D_mean_ = mean dose; D_98%_ = dose to 98% of target volume; D_2%_ = dose to 2% of target volume; D_0.03cc_ = maximum dose at 0.03 cc; D_1cc_ = maximum dose at 1 cc

From a plan quality perspective, all plans had adequate target coverage (Table 2). The mean dose to the tumour volume, D_2%_ and D_98%_ were very similar across all groups. The mean RTOG CI for DCAT, IMRT and VMAT was 1.6 (SD 0.2), 1.9 (0.4) and 1.5 (0.2), respectively. The mean Paddick CI for DCAT, IMRT and VMAT was 0.66 (0.089), 0.56 (0.079) and 0.69 (0.079), respectively. The difference between the mean Paddick and RTOG CIs of the DCAT and VMAT plans was not statistically significant but were superior to IMRT (Tables 2 and 3). The DCAT plans achieved better GI, with a mean of 3.0 (0.5) compared with 3.4 (0.5) for IMRT and 3.4 (0.4) for VMAT, and this difference was statistically significant (*P* < 0.0001).

**Table 3:** Mean difference in plan quality and OAR dosimetric parameters between treatment techniques

Parameter	Technique	Mean difference (95% CI)	Comparison P value	Global P value
Conformity index (RTOG)	DCAT vs IMRT	− 0.3 (− 0.4, − 0.1)	0.0002	
	DCAT vs VMAT	0.1 (− 0.04, 0.3)	0.14	< .0001
	IMRT vs VMAT	0.4 (0.3, 0.5)	< .0001	
Conformity index (Paddick)	DCAT vs IMRT	0.098 (0.056, 0.14)	< .0001	
	DCAT vs VMAT	− 0.027 (− 0.070, 0.016)	0.21	< .0001
	IMRT vs VMAT	− 0.13 (− 0.17, − 0.083)	< .0001	
Gradient index	DCAT vs IMRT	− 0.4 (− 0.6, − 0.3)	< .0001	
	DCAT vs VMAT	− 0.4 (− 0.6, − 0.3)	< .0001	< .0001
	IMRT vs VMAT	0.01 (− 0.2, 0.2)	0.89	
Brainstem D_1cc_ (Gy)	DCAT vs IMRT	0.5 (0.1, 1.0)	0.030	
	DCAT vs VMAT	1.1 (0.6 1.5)	< .0001	< .0001
	IMRT vs VMAT	0.6 (0.1, 1.0)	0.016	
Cochlea D_0.03cc_ (Gy)	DCAT vs IMRT	− 0.1 (− 0.8, 0.5)	0.64	
	DCAT vs VMAT	2.4 (1.8, 3.0)	< .0001	< .0001
	IMRT vs VMAT	2.5 (2.0, 3.2)	< .0001	
Cochlea D_mean_ (Gy)	DCAT vs IMRT	0.2 (− 0.4, 0.7)	0.48	
	DCAT vs VMAT	2.3 (1.8, 2.9)	< .0001	< .0001
	IMRT vs VMAT	2.1 (1.6, 2.7)	< .0001	

D_1cc_ = maximum dose at 1 cc; D_0.03cc_ = maximum dose at 0.03 cc; D_mean_ = mean dose

VMAT was superior in sparing the OARs (brainstem and cochlea). For VMAT, the mean brainstem D_max_ (D_0.03cc_), cochlear D_mean_ and cochlear D_max_ (D_0.03cc_) were 8.3 Gy (4.2), 3.4 Gy (1.7) and 3.6 Gy (1.8), respectively. The difference between all cochlear dosimetric parameters was statistically superior in favour of VMAT. The mean cochlear dose was 2.3 and 2.1 Gy lower for VMAT than DCAT and IMRT, respectively. The mean cochlear D_max_ (D_0.03cc_) was 2.4 and 2.5 Gy lower for VMAT than DCAT and IMRT, respectively. Additionally, the mean brainstem D_1cc_ was 1.1 and 0.6 Gy lower for VMAT than DCAT and IMRT, respectively (Tables 2 and 3).

The linear mixed-effects model did not show any significant association between cochlear dosimetric parameters and tumour volume or dimensions but did show significant association with the fundus distance (Table 4). Hence, linear regression modelling was used to test the association between cochlear dose parameters and the fundus distance for each treatment technique (Table 5). The results showed statistically significant associations regardless of the treatment technique. For DCAT, IMRT and VMAT, for every 1 mm increase in fundus distance, the cochlear D_max_ and D_mean_ decreased by 0.59 and 0.54 Gy, 1.0 and 0.92 Gy, and 0.73 and 0.67 Gy, respectively (Table 5).

**Table 4:** Mean difference in cochlear D_0.03cc_ and cochlear D_mean_ by tumour characteristics

Parameter	Characteristic	Mean difference (95% CI)	Global P value
Cochlea D_0.03cc_ (Gy)	Tumour volume (cc)	− 0.49 (− 1.7,0.74)	0.43
	y-diameter (mm)	0.04 (− 0.27,0.36)	0.78
	x-diameter (mm)	0.16 (− 0.28,0.60)	0.47
	Distance from fundus (mm)	− 0.69 (− 1.1,− 0.28)	0.0017
Cochlea D_mean_ (Gy)	Tumour volume (cc)	− 0.54 (− 1.7,0.59)	0.34
	y-diameter (mm)	0.05 (− 0.24,0.34)	0.73
	x-diameter (mm)	0.18 (− 0.23,0.58)	0.39
	Distance from fundus (mm)	− 0.62 (− 1.0,-0.24)	0.0023

D_0.03cc_ = maximum dose at 0.03 cc; D_mean_ = mean dose

**Table 5:** Subgroup analysis by treatment technique: mean difference in cochlear D_0.03cc_ and cochlear D_mean_ by distance from fundus

Technique	Cochlear dosimetric parameter	Mean difference per distance (Gy/mm) (95% CI)	Global P value
DCAT	Cochlea D_0.03cc_	− 0.59 (− 0.95, − 0.22)	0.0035
IMRT	Cochlea D_0.03cc_	− 1.0 (− 1.44, − 0.57)	0.0002
VMAT	Cochlea D_0.03cc_	− 0.73 (− 1.04, − 0.43)	0.0001
DCAT	Cochlea D_mean_	− 0.54 (− 0.89, − 0.20)	0.0038
IMRT	Cochlea D_mean_	− 0.92 (− 1.30, − 0.54)	0.0001
VMAT	Cochlea D_mean_	− 0.67 (− 0.95, − 0.38)	0.0001

D_0.03cc_ = maximum dose at 0.03 cc; D_mean_ = mean dose

## Discussion

Dose to the cochlea is increasingly recognised as likely a critical factor in preventing hearing loss [7–11]. However, cochlear dose sparing can be challenging since vestibular schwannomas are very often anatomically closely related to the cochlea. Indeed, of the various tumour characteristics in our study, the distance from the fundus was the only significant predictor of the cochlear dose regardless of the treatment technique, which is not surprising as the fundus distance is a surrogate for the tumour's proximity to the cochlea and will be inversely related to the cochlear dose. For SRS, although not supported by strong evidence, the current recommended cochlear dose limit is 4 Gy [18, 19]. Even for the currently recommended marginal dose of 11–14 Gy [3, 20, 21], it is still a significant challenge to restrict the dose to cochlea < 4 Gy. Moreover, for better preservation of hearing, restricting cochlear dose as low as < 3 Gy has been proposed [22]. Restricting the dose to the cochlea to such a low dose can be achieved only by means of a very steep dose gradient.

On the other hand, minimizing cochlear dose may not, by itself, be sufficient to preserve hearing as there are other factors that play a role in hearing outcome after SRS [23]. Dose to the cochlear nerve, transient tumour expansion and vascular injury from radiotherapy have all been speculated to contribute to the hearing loss. Hence, cochlear dose sparing at the expense of tumour coverage should be generally avoided. A practical approach will be to maintain tumour coverage and apply the ALARA principle to restrict dose to cochlea. Herein lies the importance of choosing the best treatment technique that facilitates maximum dose reduction to the cochlea and optimum tumour coverage.

The published dosimetric studies have mostly compared LINAC-based SRS with Gamma Knife SRS [24, 25]. We are not aware of any other study that compared all three DCAT, IMRT and VMAT techniques for LINAC-based SRS for VS, particularly from the standpoint of cochlear dose sparing. This comparison is very relevant as many centres have access to only LINAC-based SRS, with increasing availability of IMRT and VMAT techniques to deliver the treatment.

We were able to show that, although each technique generated high-quality plans that met the planning objectives, VMAT plans were superior to DCAT or IMRT. VMAT produced a more conformal plan than IMRT and was on par with DCAT. However, the superiority of VMAT was unequivocal in sparing OAR as it delivered the lowest brainstem and cochlear doses. Compared to IMRT and DCAT, the mean difference between cochlear dosimetric parameters was at least 2 Gy less for VMAT. We believe such a decrease may be of substantial advantage when the aim is to achieve maximum dose sparing to the cochlea. DCAT was able to achieve better GI compared to VMAT, but this by itself is of minimal significance since the higher GI for VMAT is likely a trade-off for achieving better OAR dose sparing.

Balik et al. [25] compared GK and VMAT plans for eight VS patients, prescribing 12–13 Gy, and showed no significant difference in plan quality parameters or OAR doses. In this study, the cochlear D_mean_ was 7.23 (3.13) Gy for VMAT plans, which is higher than the 3.4 (1.7) Gy in our study, probably due to the planning dose constraint they applied to cochlea (D_max_ of 12 Gy). Abacioglu et al. [26] compared GK and RapidArc (TrueBeam Linac) SRS plans for 6 VS cases and prescribed 12.5 Gy. Of the two plan optimisation strategies they used, the second strategy is of interest since this aimed for maximum OAR sparing. The cochlear D_mean_ was 3.3 (0.8) and 4.1 (0.9) for GK and RapidArc, respectively. Our VMAT plans achieved a slightly lower cochlear D_mean_, but it is comparable given our lower marginal dose of 12 Gy. A larger study by Kim et al. [24], comparing GK and VMAT plans of 19 patients, also did not show any significant difference in the cochlear doses. They showed that VMAT using 5 coplanar arcs produced superior Paddick CI for targets larger than 0.5 cc compared to GK, but the mean cochlear dose (6.0 [1.4] Gy) was higher. Their prescription dose was 12 Gy and they also used a maximum dose of 12 Gy as the cochlear dose constraint. Dutta et al. [27] compared DCAT (Brainlab) with CyberKnife and prescribed 13–15 Gy. They found that DCAT delivered a significantly higher cochlear D_mean_ compared to CyberKnife (6.9 [0.7] vs 5.4 [0.6]). Our study also demonstrated that VMAT reduced the mean cochlear dose by at least 2 Gy compared to DCAT and delivered lower cochlear D_mean_ than this study. This supports the use of VMAT in institutions that do not have access to CyberKnife, although further studies comparing these two techniques may quantify this.

Sharma et al. [28] created 5 different sets of static conformal field, DCAT and IMRT SRS plans for their cohort of 8 patients and, of these, the two DCAT and IMRT plans that prescribed 12 Gy to the 80% isodose line are of interest here. Unlike our study, IMRT was able to significantly lower the mean cochlear dose (5.0 [0.98] Gy), but not the maximum cochlear dose (9.8 [1.8] Gy), compared with DCAT. While their mean dose was slightly lower than that achieved in our IMRT plans (5.5 [2.3] Gy), we could not compare the maximum doses as they did not mention how the maximum dose was defined.

In contrast to our findings, Lagerwaald et al. [29] failed to show any difference in reducing the maximum dose to critical organs, including the cochlea and brainstem, when they compared VMAT with single arc and 5 arc DCAT for three cases. A plausible explanation for this failure of VMAT to achieve superior sparing of critical organs could be due to tumour location in the small sample. Our study showed that distance from fundus was a significant predictor of the ability to spare the cochlea regardless of the treatment technique. The 18 cases we selected may have increased the variability of tumour size and location, and thereby allowed the benefits of VMAT to be more evident.

Our study has some limitations. First, the VMAT planning was performed using newer planning software than that used to produce DCAT and IMRT plans. Second, we did not set a cochlear dose constraint, which could have achieved better results, not only for IMRT and DCAT but also for VMAT; we chose not to do this in order to maintain uniformity between treatment planning techniques and to allow maximum dose sparing with least risk of compromising tumour coverage. Lastly, although we demonstrated the dosimetric superiority of VMAT, we did not compare other factors like planning time and treatment delivery time.

## Conclusion

Our study supports the use of VMAT as the preferred technique for LINAC-based SRS for vestibular schwannoma where hearing preservation is a goal. LINAC-based SRS treatment planning using the VMAT technique (Brainlab) can achieve better cochlear dose sparing than DCAT or IMRT while maintaining tumour coverage.

## Data Availability

Research data are stored in an institutional repository and will be shared upon reasonable request to the corresponding author.

## Abbreviations

SRS: Stereotactic radiosurgery
VS: Vestibular schwannoma
DCAT: Dynamic conformal arc therapy
IMRT: Intensity modulated radiation therapy
VMAT: Volumetric modulated arc therapy
OAR: Organs-at-risk
PTA: Pure-tone average
GTV: Gross tumour volume
PTV: Planning target volume
CI: Conformity index
GI: Gradient index
